# Sewage treatment plant associated genetic differentiation in the blue mussel from the Baltic Sea and Swedish west coast

**DOI:** 10.7717/peerj.2628

**Published:** 2016-10-27

**Authors:** Josefine Larsson, Mikael Lönn, Emma E. Lind, Justyna Świeżak, Katarzyna Smolarz, Mats Grahn

**Affiliations:** 1 School of Natural Science, Technology and Environmental Studies, Södertörn University, Huddinge, Stockholm, Sweden; 2 Department of Aquatic Resources, Swedish University of Agricultural Sciences, Drottningholm, Stockholm, Stockholm, Sweden; 3 Department of Marine Ecosystem Functioning, University of Gdansk, Institute of Oceanography, Gdynia, Poland

**Keywords:** Baltic Sea, Blue mussel, Environmental pollution, AFLP, Sewage treatment effluents, Harbor, Genetic differentiation

## Abstract

Human-derived environmental pollutants and nutrients that reach the aquatic environment through sewage effluents, agricultural and industrial processes are constantly contributing to environmental changes that serve as drivers for adaptive responses and evolutionary changes in many taxa. In this study, we examined how two types of point sources of aquatic environmental pollution, harbors and sewage treatment plants, affect gene diversity and genetic differentiation in the blue mussel in the Baltic Sea area and off the Swedish west coast (Skagerrak). Reference sites (REF) were geographically paired with sites from sewage treatments plant (STP) and harbors (HAR) with a nested sampling scheme, and genetic differentiation was evaluated using a high-resolution marker amplified fragment length polymorphism (AFLP). This study showed that genetic composition in the Baltic Sea blue mussel was associated with exposure to sewage treatment plant effluents. In addition, mussel populations from harbors were genetically divergent, in contrast to the sewage treatment plant populations, suggesting that there is an effect of pollution from harbors but that the direction is divergent and site specific, while the pollution effect from sewage treatment plants on the genetic composition of blue mussel populations acts in the same direction in the investigated sites.

## Introduction

Human activities are constantly contributing to environmental changes that serve as drivers for evolutionary responses in many taxa (see review by Palumbi, 2001 and Smith & Bernatchez, 2008). Natural populations are expected to experience changes in genetic diversity and genetic differentiation through genetic drift, gene flow and selection, and it is evident that anthropogenic activities can have impact on these processes (Smith & Bernatchez, 2008; Banks et al., 2013). Anthropogenic environmental pollutants in the aquatic environment originate from, for example, sewage effluents, agriculture and industrial processes (Walker et al., 2006). Pollutants can affect organisms on an individual level by affecting their behavior and/or physiology, but also on a population level and a multigenerational level trough natural selection (Chevin, Lande & Mace, 2010; Banks et al., 2013; Whitehead, 2014). In populations with strong gene flow (panmixia or near panmixia) and random larval dispersal across habitats, heritable trans-generational local adaptation is hindered, but footprints of spatially varying single-generation selection across habitats can still be detected (e.g., in the European eel (*Anguilla anguilla*) and American eel (*Anguilla rostrata*) (Gagnaire et al., 2012; Ulrik et al., 2014; Laporte et al., 2016).

**Figure 1:**
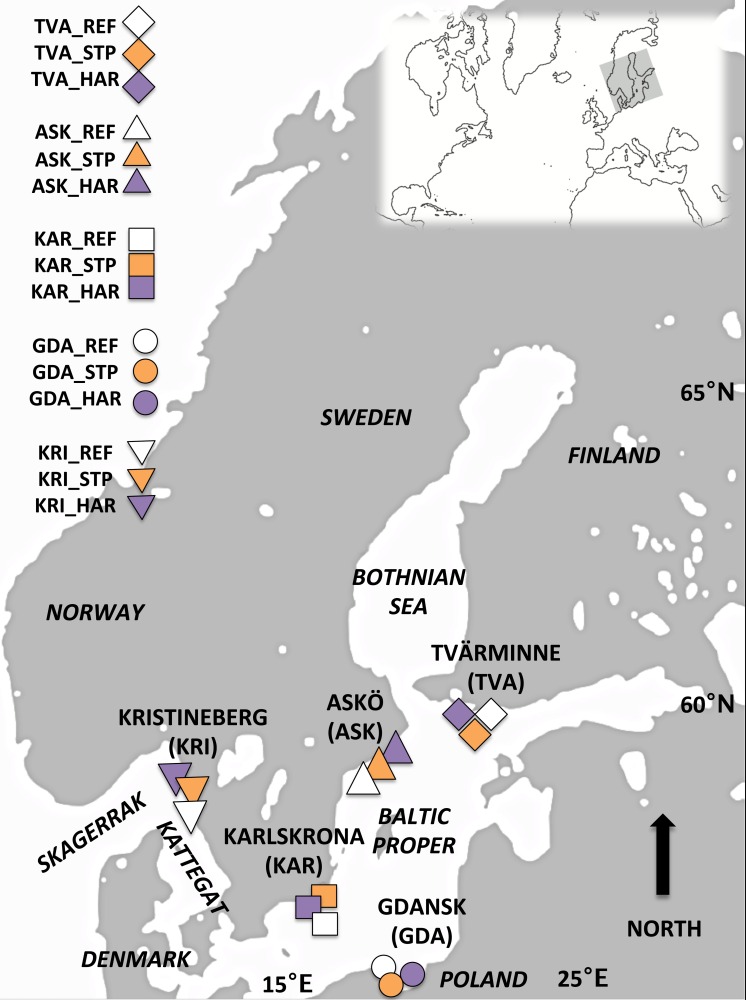
Map of the study area. Sampling locations for Blue mussels within the Baltic Proper and off the Swedish West Coast: Askö (ASK), Tvärminne (TVA), Karlskrona (KAR), Gdansk (GDA) and Kristinberg (KRI). At each location three sites representing different pollution types were sampled; reference (REF) sites, sewage treatment plant (STP) effluent affected sites, and sites in the vicinity of harbors (HAR).

The Baltic Sea is one of the largest brackish water bodies in the world, with a salinity gradient spanning from 17 to 25 along the Swedish west coast, 5.5 to 7.3 in the Baltic Proper, approximately 5 in the Bothnian Sea and 2 to 4 in the Bothnian Bay (Elmgren, 2001; Zillén et al., 2008) (Fig. 1). One of the organisms in the Baltic Sea that has successfully adapted to the brackish environment is the blue mussel (*Mytilus*) and the adaptation to brackish water has caused morphological and genetic differentiation between the blue mussels off the Swedish west coast and blue mussels in the Baltic Sea (Johannesson, Kautsky & Tedengren, 1990; Johannesson & Andre, 2006). The genetic differentiation between population from the Baltic Sea and the west coast can be explained by the salinity cline and the low connectivity between the regions, and is paralleled by the distribution of lineages from two sister species of *Mytilus*, blue mussels (Larsson et al., in press). Clear differences at multiple allozyme characters have been found between the blue mussels in the Baltic Sea and North Sea with a cline in the Öresund region (Theisen, 1978; Väinölä & Hvilsom, 1991), attributed to differences originating from *M. edulis* in the North Sea and *M. trossulus* in the Baltic Sea. However, in other genetic characters the Baltic population appears to be strongly introgressed by parts of the *M. edulis* genome (Stuckas et al., 2009; Kijewski et al., 2011; Väinölä & Strelkov, 2011; Zbawicka et al., 2012). The blue mussel in the Baltic Sea is best described as a species complex, *Mytilus edulis trossulus* with extensive mixing of the *M. edulis* and *M. trossulus* genomes (Väinölä & Strelkov, 2011). The genetic differentiation among sites within the Baltic Proper is very low, indicative of strong gene flow, (Johannesson & Andre, 2006; Larsson et al., in press) and can be attributed to the long planktonic larval phase (5–6 weeks) (Kautsky, 1982), and the high oceanographic connectivity within the Baltic Proper (Larsson et al., in press).

The blue mussel (*M. edulis trossulus*), is a keystone species in the Baltic Sea ecosystem serving several ecological important features such as filtering the water and providing a food-chain link between the benthos and the pelagic (Kautsky, & Evans, 1987). The blue mussel is a habitat-forming species and serves as an important food source for many fish and bird species (Koivisto, 2011). It is also commonly used as a bioindicator organism in monitoring of marine pollution (Viarengo & Canesi, 1991).

One of the main point sources of environmental pollution in the aquatic environment are sewage treatment plants (STP) (Walker et al., 2006). Wastewater from industry and households are processed by sewage treatment plants and even though the efficiency of the sewage treatment plants has increased over the last 20 years sewage effluents are still considered a main source of pollution in the aquatic environment (Bolong et al., 2009; HELCOM, 2010). Many compounds, for example pharmaceuticals, personal care product residues, surfactants (Rosi-Marshall & Royer, 2012), phthalates, perflourinated compounds (PFCs) and even persistent organic pollutants (POPs) are not fully eliminated during the cleaning process (Rule et al., 2006; Bolong et al., 2009). Sewage effluents have been shown to have several negative biological and endocrine disruptive effects on organisms including; immunological (Akaishi et al., 2007), genotoxic and lysosomal responses (Turja et al., 2015), feminization in freshwater mussels (*Elliptio complanata*) (Gagné et al., 2011), feminization and increased intersex in roaches (*Rutilus rutilus*) (Jobling et al., 1998) and up-regulation of vitellogenin in males both in freshwater mussels (*E. complanata*) (Bouchard et al., 2009; Gagné et al., 2011), marine mussels (*Mytilus galloprovincialis*) (De los Ríos et al., 2013) and sticklebacks (*Gasterosteus aculeatus*) (Björkblom et al., 2013).

Only a few studies have addressed the long term and evolutionary effects of sewage treatment plant effluents on wild populations (but see Puritz & Toonen, 2011; Wedekind, 2014; Hamilton et al., 2014). Puritz & Toonen (2011) showed that despite high genetic connectivity among bat stars (*Patiria miniata*) in the Southern California Bight, the genetic structure as estimated by mtDNA and microsatellites was correlated with runoff from sewage treatment plants and storm water. The runoff from sewage treatment plants and storm water was proposed to cause larval mortality and act as a barrier to larval dispersal, resulting in genetic differentiation among populations. In a study of roaches (*R. rutilus*) living in rivers highly polluted by sewage effluents, Hamilton and colleagues (2014), showed strong pollution effects of sewage effluents with a high feminization rate of males, however they did not find any pollution associated population genetic effects (see Wedekind, 2014) (Hamilton et al., 2014). Wedekind (2014) stressed the importance of further studies on the evolutionary effects of sewage effluents.

Persistent pollution “hot spots” in the marine environment include harbors and ship wrecks (HAR). These are often highly affected by high concentrations of heavy metals, hydrocarbons, polycyclic aromatic hydrocarbons, polychlorinated biphenyls, polychlorinated benzodioxins, polychlorinated dibenzofurans, polybrominated biphenyls and dioxins (Ma, Cowles & Carter, 2000; Turja et al., 2013; Turja et al., 2014). These substances are known to have genotoxic, mutagenic, carcinogenic and/or negative physiological effects on living organisms (Pérez-Cadahía et al., 2004; Walker et al., 2006; Smolarz & Berger, 2009). In several genetic studies of aquatic organisms exposed to heavy metals (see review by Mussali-Galante et al., 2014) and industrial sites/harbors (Ma, Cowles & Carter, 2000; Williams & Oleksiak, 2011; Giantsis, Kravva & Apostolidis, 2012; Bach & Dahllöf, 2012) genetic differentiation and/or difference in gene diversity between pollution affected sites and reference sites have been identified. In contrast, a genetic study of the mussel *M. galloprovincialis* from the Adriatic Sea showed no genetic differentiation between highly polluted and reference sites and higher gene diversity at the polluted sites suggesting that high levels of gene flow in the mussels may conceal traces of local genetic processes (Štambuk et al., 2013). High gene flow is common in mussels and has also been shown in blue mussels in the Baltic Sea (Larsson et al., in press).

Contamination of aquatic environments from point sources like sewage treatment plants and harbors typically include a range of pollutants that have different effects on organisms, therefore a complex selective response including multiple genes is expected (Laporte et al., 2016). Neutral genetic markers, for example microsatellites, mainly provide insights about neutral processes such as reduced gene flow and breaks in connectivity. Genetic methods based on genome wide markers, for example amplified fragment length polymorphism (AFLP), cover a larger scope of markers for both neutral and selective processes and they can provide a more comprehensive description of contemporary selective processes (Stinchcombe & Hoekstra, 2008; Williams & Oleksiak, 2008). In addition, AFLP provides an opportunity to identify loci linked to adaptive variation among habitats (Williams & Oleksiak, 2008; Nosil, Funk & Ortiz-Barrientos, 2009; Lind & Grahn, 2011). Detection of loci deviating from random expectations using AFLP has been shown to give similar rates of detection as by using single nucleotide polymorphism (SNP) chips (Gomes et al., 2015).

The aim of this study was to explore how two types of point sources of environmental pollution (STP and HAR) affect gene diversity and genetic differentiation in the blue mussel in the Baltic Sea and the Swedish west coast (Skagerrak). By using a combination of a nested sampling scheme (including sites from reference habitats, geographically paired with sites from sewage treatments plant and harbors), with a high-resolution marker (AFLP), we examined if pollution from sewage treatment plants and harbors could (i) affect the gene diversity at polluted sites, (ii) cause genetic differentiation between polluted sites and reference sites, and (iii) cause differentiation that is consistent in direction in sites within pollution types or if the direction of differentiation is divergent between individual polluted sites. We also aimed at evaluating the effect of the species introgression pattern on the genome wide genetic structure and effects in combination with pollution type.

## Materials and Methods

### Sampling

Mussels were sampled during June 2012 and 2013 from five locations in the Baltic Proper (BP) and off the Swedish west coast (WC): Trosa/Nynäshamn Archipelago (east coast of Sweden) (ASK), Tvärminne in the Gulf of Finland (TVA), Karlskrona (Southern Sweden) (KAR), the Gulf of Gdańsk, Poland (GDA) and Kristineberg (the Swedish west coast, Skagerrak) (KRI) (Fig. 1, Table 1). Within each location three sites representing different pollution types were chosen based on information from reports and local authorities’ assessment schemes. These sites were located close to a sewage treatment plant (STP) effluent, the vicinity of a harbor (HAR), and a reference (REF). The sewage treatment plants studied include large plants with >100,000 persons connected to the plant (ASK_STP and GDA_STP), a medium plant >40,000 persons (KAR_STP) and two small plants each with approximately 7,000 persons connected (TVA_STP and KRI_STP), but all STP sites had similar amounts of nitrogen and phosphorus emission (see Table S1 for details). The sampled vicinities of harbors included different types of harbors; ferry ports (ASK_HAR, TVA_HAR), cargo and ferry Gdynia harbor (GDA_HAR), a refinery port at Preemraff (KRI_HAR), and a naval port (KAR_HAR) (see Table S2 for details). STP and HAR sites were paired with a reference site (REF) that was not influenced by sewage effluents or harbor associated pollutants. All REF sites were situated within 5–30 km from the STP and HAR sites (Tables 1 and 2). The geographical distances between sites (Table 2) were estimated using the most direct marine route in Google Earth version 7.1.2.2014 (Google, Mountain View, CA, USA). Temperature regimes and salinity differed between the different locations (west coast, and southern and northern Baltic Proper) but were similar among the three sampled sites within each of the locations (Table 1).

**Table 1:** Sampling and species data. Sampling locations with abbreviations, number of individuals per site (*N*), gene diversity (***H***_***E***_) with standard error (S.E), salinity, geographical coordinates, percentage of individuals with species identity (based on the Glu-5′ marker) *M. edulis* (0), *M. trossulus* (2) and heterozygotes between *M. edulis* and *M. trossulus* (1). Mean gene diversity is given for each pollution type.

Location	Pollution type	N	*H*_*E*_ S.E	Salinity	Coordinates	Species identity 0/1/2 (%)	Pollution type (*H*_*E*_)
Askö (ASK)	REF	26	0.12273 0.00728	6.2	58°48.31′N 17°38.91′E	24/56/20	REF $\overline{{\mathbi{H}}_{\mathbi{E}}}=0.131$
Tvärminne (TVA)	REF	27	0.12108 0.00746	5.5	59°49.69′N 23°15.27′E	31/46/23	
Karlskrona (KAR)	REF	27	0.13694 0.00773	7.2	56°06.43′N 15°33.98′E	56/37/6	
Gdansk (GDA)	REF	30	0.13158 0.00747	7.3	54°29.37′N 18°38.60′E	50/36/14	
Kristineberg (KRI)	REF	28	0.14312 0.00756	23	58°14.78′N 11°26.16′E	96/4/0	
Askö (ASK)	STP	25	0.14339 0.00703	5.6	58°02.47′N 17°41.74′E	29/33/38	STP $\overline{{\mathbi{H}}_{\mathbi{E}}}=0.130$
Tvärminne (TVA)	STP	24	0.13038 0.00753	5.5	59°48.40′N 23°00.94′E	33/58/8	
Karlskrona (KAR)	STP	25	0.12455 0.00747	6.9	56°09.34′N 15°37.18′E	31/50/19	
Gdansk (GDA)	STP	28	0.12014 0.00736	7.3	54°35.98′N 18°32.83′E	36/32/32	
Kristineberg (KRI)	STP	26	0.12629 0.00705	26	58°17.12′N 11°26.02′E	100/0/0	
Askö ( ASK)	HAR	29	0.12964 0.00726	5.9	58°54.54′N 17°58.22′E	53/40/7	HAR $\overline{{\mathbi{H}}_{\mathbi{E}}}=0.124$
Tvärminne (TVA)	HAR	25	0.12691 0.00743	5.5	59°49.16′N 22°56.64′E	38/58/4	
Karlskrona (KAR)	HAR	24	0.12642 0.00761	6.9	56°08.83′N 15°35.36′E	43/38/19	
Gdansk (GDA)	HAR	24	0.11590 0.00737	7.3	54°33.00′N 18°36.00′E	54/33/13	
Kristineberg (KRI)	HAR	27	0.12258 0.00715	25	58°20.90′N 11°24.60′E	96/4/0	

**Table 2:** Pairwise genetic differentiation *F*_*ST*_. Pairwise genetic differentiation *F*_*ST*_ (below diagonal) between all sites and pairwise geographical distances (km) between all sites measured as the shortest possible way in water (above diagonal). Significant pairwise *F*_*ST*_ differences after are indicated with light grey and significant pairwise *F*_*ST*_ differences after false discovery rate (FDR) correction are indicated with dark grey (FDR = 0.05).

	ASK_HAR	ASK_REF	ASK_STP	TVA_HAR	TVA_REF	TVA_STP	KAR_HAR	KAR_REF	KAR_STP	GDA_HAR	GDA_REF	GDA_STP	KRI_HAR	KRI_REF	KRI_STP
ASK_HAR	–	30	55	315	330	315	390	390	380	510	495	515	940	925	930
ASK_REF	0.0000	–	30	335	350	340	370	365	370	490	485	500	925	910	915
ASK_STP	0.0019	0.0028	–	345	360	350	250	245	350	510	350	510	950	935	940
TVA_HAR	0.0000	0.0000	0.0000	–	20	5	640	635	640	650	650	650	1155	1170	1180
TVA_REF	0.0000	0.0000	0.0028	0.0000	–	15	555	650	660	660	660	650	1200	1190	1200
TVA_STP	0.0000	0.0000	0.0000	0.0000	0.0000	–	645	640	645	650	650	640	1195	1175	1185
KAR_HAR	0.0057	0.0030	0.0063	0.0020	0.0052	0.0012	–	10	5	290	290	295	575	560	565
KAR_REF	0.0041	0.0022	0.0090	0.0012	0.0034	0.0022	0.0000	–	10	280	285	290	565	550	555
KAR_STP	0.0028	0.0026	0.0035	0.0000	0.0037	0.0001	0.0000	0.0002	–	290	210	285	575	560	560
GDA_HAR	0.0010	0.0000	0.0056	0.0000	0.0000	0.0015	0.0021	0.0009	0.0013	–	10	10	790	770	780
GDA_REF	0.0013	0.0011	0.0056	0.0000	0.0000	0.0010	0.0000	0.0000	0.0000	0.0000	–	15	700	770	775
GDA_STP	0.0029	0.0009	0.0057	0.0000	0.0000	0.0000	0.0000	0.0003	0.0000	0.0000	0.0006	–	795	780	780
KRI_HAR	0.0704	0.0649	0.0621	0.0637	0.0799	0.0733	0.0634	0.0593	0.0530	0.0636	0.0596	0.0737	–	15	10
KRI_REF	0.0654	0.0601	0.0553	0.0609	0.0741	0.0683	0.0589	0.0553	0.0517	0.0625	0.0551	0.0690	0.0000	–	5
KRI_STP	0.0712	0.0672	0.0606	0.0683	0.0831	0.0752	0.0672	0.0663	0.0563	0.0708	0.0649	0.0778	0.0000	0.0000	–

**Notes.**

Significant prior to FDR correction.

Significant after FDR correction FDR = 0.05.

At each of 15 sites, 30 blue mussels (24–30 individuals used in the subsequent AFLP analyses, Table 1) with a mix of both sexes were sampled. The mussels were sampled using either a benthic sledge, a triangular bottom scraper or by hand, from a depth of between 1 and 13 m depending on local distribution. The age of each individual was estimated by counting growth rings (see Haskin, 1954) and all individuals included in this study had an estimated age of 2–5 years. From each individual the adductor muscle was dissected, snap frozen and immediately stored in −80 °C, prior to DNA isolation.

### DNA isolation

Total genomic DNA was isolated from a small piece of the muscle tissue ∼2 × 2 mm of each individual using the E.Z.N.A. Mollusc DNA Kit (OMEGA Bio-Tek, Norcross, GA, USA), with small modification of the manufacturers protocol. In short, tissue was incubated overnight at 56 °C in 250 µl ML1 lysis buffer and 25 µl proteinase K followed by a chloroform:isoamyl (24:1, 300 µl) extraction step with 3 min centrifugation (at 14,000 rpm). DNA suspended in the aqueous phase was carefully separated from the interphase (200-300 µl) and 300 µl MBL buffer with 10 µl RNAse was added, and samples were incubated for 10 min at 70 °C. The DNA was precipitated from the solution with 99.7% ethanol, transferred to DNA HiBind spin columns and centrifuged to remove any traces of alcohol. Purified DNA was eluted with 50 µl 10 mM Tris–HCl buffer (pH 8.5) (preheated to 70 °C). DNA quality was visually estimated using agarose gel 1.5% electrophoresis (100 V, 20–30 min) stained with (5 µl/100 ml, 30 min) SYBRSafe (Life technologies).

### Introgression analyses

Due to the complex system of occurrence of *M. edulis* and *M. trossulus* in the Baltic Sea and the west coast, and extensive mixing of the* M. edulis* and *M. trossulus* genomes (Väinölä & Strelkov, 2011) in the Baltic Sea, we used the Glu-5′ (ME 15/16) (Inoue et al., 1995) species marker to evaluate any remaining species identity effects on the genetic structure. This marker is commonly used and distinguishes between all three *Mytilus* taxa found in the studied area (Kijewski et al., 2006). Individuals were genotyped at locus Glu-5′ (ME 15/16) (Inoue et al., 1995), and each individual was coded as homozygous *M. edulis, M. trossulus, M. galloprovincialis* or as heterozygotes between them. The AFLP method is suitable for analyses of closely related species but homoplasy, the non-homology of bands with the same electrophoretic properties, is expected to be more frequent in hybrid populations (Egger et al., 2007). Occurrence of homoplasy will have four main consequences for population genetic inferences: (1) an overestimation of the frequency of the presence allele; (2) an underestimation of the differentiation between populations; (3) an overestimation or underestimation of the heterozygosis, depending on marker frequency; and (4) reduction in the power to detect selective loci (Caballero, Quesada & Rolán-Alvarez, 2008).

The Glu-5′ PCR fragments were amplified in 10 µl reactions containing: 20–40 ng DNA, 25 mM MgCl_2_, 1 µl 10X PCR-buffer, 5 U AmpliTaq (Applied Biosystems), 1 µl BSA, 10 mM dNTP (Thermo Scientific Molecular Biology), and100 µM of each primer. Forward primer (ME15) 5′-CCAGTATACAAACCTGTGAAGA-3′ 5′-end was labeled with fluorescein and reverse (ME16) 5′-TGTTGTCTTAATAGGTTTGTAAGA-3′ (MWG Biotech). The amplification cycle consisted of an initial denaturation for 5 min at 94 °C followed by 38 cycles at 94 °C for 30 s, 55 °C for 30 s and 72 °C for 45 s, followed by elongation for 10 min at 72 °C. DNA fragments were separated on an ABI-3730XL capillary electrophoreses unit at Uppsala Genome Center with separation medium POP7™ Polymer (Applied Biosystems), size standard GeneScan™ 500 ROX™ (Applied Biosystems), injection time 15 s (1.6kV), run time 1,600 s and array length 50 cm.

### Generation of AFLP markers

Markers were generated as described by Vos et al. (1995) with minor modifications as described by Bensch et al. (2002). Briefly, genomic DNA (20–40 ng/ml) was digested with restriction enzymes for 1h at 37 °C. Each 20 µl reaction contained 0.5 µl of 50U EcoR1 (Thermo Scientific Molecular Biology) and 0.5 µl of 50U Tru1 (Thermo Scientific Molecular Biology), 2 µl 10x TA-buffer (Thermo Scientific Molecular Biology), 1 µg BSA and ddH_2_O. Followed by ligation for 3 h at 37 °C, in; ligation buffer (10X), 100 mM E-adaptor 5′-CTCGTAGACTGCGTACC-3′, 3′-CATCTGACGCATGGTTAA-5′ (MWG Biotech), 100 mM M-adaptor 5′-GACGATGAGTCCTGAG-3′, 3′-TACTCAGGACTCAT-5′  (MWG Biotech) and 10 U/ml T4 ligase (Thermo Scientific Molecular Biology).

Pre-amplification was performed in 20 µl reactions containing 10 µl DNA template (cut, ligated and diluted), 100 µM E-primer 5′-GACTGCGTACCAATTCA-3′ and 100 µM M-primer 5′-GATGAGTCCTGAGTAAC-3′, 25 mM MgCl_2_, 2 µl PCR-buffer (10x), 0.8 µl BSA, 1 mM dNTP, 5U Taq polymerase and ddH_2_O. Pre-amplification PCR conditions included an initial denaturation step for 2 min at 94 °C followed by 20 cycles of 94 °C for 30 s, 56 °C for 30 s and 72 °C for 60 s, and a terminal step at 72 °C for 10 min.

For selective amplification eleven primer combinations were evaluated using a subset of individuals from all the geographical regions and habitats. The two chosen combinations M**CTG**_E**AGC** and M**CTT**_E**ACT,** showed polymorphic bands, with a congruent pattern in duplicated individuals. The PCR reaction for selective amplification was conducted containing; 2.5 µl of the diluted pre-PCR template, 25 mM MgCl_2_, 1 µl 10x PCR-buffer, 1 µl BSA, 1 mM dNTP, 100 mM E-primer 5′-GACTGCGTACCAATTCNNN-3′ with a fluorescein 5′-end, 100 mM M-primer 5′-GATGAGTCCTGAGTAANNN-3′ and 5U Taq polymerase and ddH_2_O to a volume of 10 µl. The selective amplification cycle consisted of denaturation for 2 min at 94 °C, 12 cycles at 94 °C for 30 s, 56 °C with a touch-down temperature −0.7 °C for 30 s and 72 °C for 60 s, followed by 23 cycles at 94 °C for 30 s, 56 °C for 30 s, 72 °C for 60 s and a terminal step at 72 °C for 10 min.

The DNA fragments (AFLP) were separated on an ABI-3730XL capillary electrophoresis unit at Uppsala Genome Center with separation medium POP7™ Polymer (Applied Biosystems), size standard GeneScan™ 500 ROX™ (Applied Biosystems), injection time 15 s (1.6kV), run time 1,600 s and array length 50 cm.

### AFLP analyses

To generate a genotype matrix for each primer combination, AFLP data from the two primer combinations were analyzed separately in Genemapper 4.0 (Applied Biosystems). Analyses settings: primer M**CTG**_E**AGC**: 250–500 bp, (Primer M**CTT**_E**ACT**: 230–500 bp), bin width 1.5 bp, peak detector 170 rfu, normalization using sum of signals from all individuals within one primer combination and no smoothing. For error rate analysis 7–14% of the samples were randomly chosen and duplicated from extraction and/or ligation step. The duplicates were manually checked for congruency and the recorded duplicated loci where used to calculate error rate i.e., the number of inconsistent loci per primer combination divided by total number of loci resulting in the percentage of error. The two genotype-matrices were merged into one dataset used in all subsequent analyses.

### Introgression and species identity analyses

To score the target allele length at the Glu-5′ locus, GeneMarker 2.5.2 (SoftGenetics LCC) software with size standard GS500 and analysis type Fragment (Animal) was used. In total, 364 individuals were scored, by the allele lengths as *M. galloprovincialis* (allele length 124 bp), *M. trossulus* (165 bp) or *M. edulis* (177 bp) Twenty percent of the samples were duplicated to check for congruency.

### Statistical analyses

#### Gene diversity and genetic differentiation

Estimates of genetic diversity within sites (*H*_*E*_) and between sites (*F*_*ST*_) were obtained using the software AFLP-SURV 1.0 (Vekemans, 2002) with the approach of Lynch & Milligan (1994), assuming Hardy-Weinberg equilibrium and using the Bayesian method with a non-uniform prior distribution of allele frequencies (Zhivotovsky, 1999) and 10,000 permutations. Tests of difference in gene diversity between the sampled pollution types both including (*N*_REF_ = 5, *N*_STP_ = 5, *N*_HAR_ = 5) and excluding the west coast sites (*N*_REF_ = 4, *N*_STP_ = 4, *N*_HAR_ = 4), were performed as ANOVAs in the Rcmdr package (Fox, 2005) with post-hoc tests using the multcomp package (Hothorn, Bretz & Westfall, 2008) in R. 3.1.3 (R Development Core Team, 2015).

Genetic differentiation, *F*_*ST*_, was calculated, in AFLP-SURV (Vekemans, 2002) using different aspects of the dataset. To examine pair-wise *F*_*ST*_ differences between all sites, pair-wise comparisons were made (Table 2). To examine *F*_*ST*_ difference between the pollution types, the sites within each pollution type were pooled (both including and excluding the west coast sites) (Table 3). To examine *F*_*ST*_ difference between the five locations (ASK, TVA, KAR, GDA and KRI) each pollution type was analyzed separately in AFLP-SURV including each location (Table 3). The significance of all *F*_*ST*_ values were based on 10,000 permutations and adjusted for multiple testing by false discovery rate (FDR) calculations (FDR = 0.05) (Benjamini, 2010) using the online FDR calculator (www.sdmproject.com/utilities/?show=FDR) for 105 pairwise comparisons, 10 locations comparisons and 2 pollution type comparisons (STP/REF, HAR/REF).

**Table 3:** Genetic differentiation, *F*_*ST*_, between pollution type and between the five locations (ASK, TVA, KAR, GDA and KRI), within each pollution type. Genetic differentiation, *F*_*ST*_, between pollution type STP/REF and HAR/REF using all sites and Baltic Proper sites only and genetic differentiation, *F*_*ST*_, between the five locations (ASK, TVA, KAR, GDA and KRI), within each pollution type. Significant differentiation after false discovery rate (FDR) correction is indicated in grey (FDR = 0.05).

Pollution type	*F*_*ST*_	FDR corrected *p*-value (0.05)
STP/REF ALL	0.017	0.0048
STP/REF BP	0.022	0.022
HAR/REF ALL	0.0005	0.685
HAR/REF BP	0.0013	0.936

Location/pollution type	*F*_*ST*_	FDR (0.05)
**STP**		
ASK_TVA	0.0025	0.5027
ASK_KAR	0.0035	0.098
ASK_GDA	0.0064	0.052
ASK_KRI	0.0604	0.000
TVA_KAR	0.0001	0.369
TVA_GDA	0.0025	0.5684
TVA_KRI	0.0752	0.000
KAR_GDA	0.0016	0.5027
KAR_KRI	0.0567	0.000
GDA_KRI	0.0774	0.000
**REF**		
ASK_TVA	0.006	0.9610
ASK_KAR	0.022	0.1648
ASK_GDA	0.0011	0.2084
ASK_KRI	0.0558	0.000
TVA_KAR	0.0034	0.1138
TVA_GDA	0.0003	0.4786
TVA_KRI	0.0736	0.000
KAR_GDA	0.0008	0.4259
KAR_KRI	0.0553	0.000
GDA_KRI	0.0550	0.000
**HAR**		
ASK_TVA	0.0030	0.7213
ASK_KAR	0.0057	0.028
ASK_GDA	0.0010	0.210
ASK_KRI	0.0705	0.000
TVA_KAR	0.0020	0.1448
TVA_GDA	0.0041	0.7213
TVA_KRI	0.0637	0.000
KAR_GDA	0.0021	0.1513
KAR_KRI	0.0634	0.000
GDA_KRI	0.0630	0.000

#### Genetic structure

The large-scale geographical component in the sampling scheme, and the complex species introgression pattern in the studied area made it necessary to take these components into account when analyzing possible effects of pollution type. As the genetic difference between the west coast and the Baltic Proper locations is expected to be large (Larsson et al., in press) and to get a higher resolution in the Baltic Proper analyses were performed on different subsets of the data; STP/REF and HAR/REF, including and excluding the west coast sites. Effects of location, species identity and pollution on the genetic structure were evaluated using constrained principal coordinate analyses (cPCoA). The cPCoA is a supervised ordination model, where location, species identity and pollution were used as explanatory variable. The cPCoA as implemented in the capscale procedure (R package vegan, Oksanen et al., 2015) is equivalent to redundancy analysis (RDA) but allows other distance measures than Euclidian, we used Jaccard distances, which are more suitable for binary data (e.g., AFLP data). The significance of the explanatory variable was assessed using a permutation test, i.e., permutation based ANOVA as implemented in the vegan package (Oksanen et al., 2015) in R 3.2.1 (R Development Core Team, 2015). This was done for all sites (and all pollution types together) both including and excluding the west coast.

To understand the general species effect on the genetic structure (i.e., the AFLP matrix) a cPCoA was conducted using all sites (and all pollution types together) both including and excluding the west coast, with species identity as explanatory variable.

To test the effect of pollution type on the genetic structure (i.e., the AFLP matrix) cPCoA models including location, species identity, pollution type and the interaction between pollution types and species were constructed. Here location, species identity and pollution type were used as explanatory variables in an order dependent ANOVA model performed by using the setting “by terms” as implemented in the vegan package (Oksanen et al., 2015). The term location (in the order dependent ANOVA model) measures the simultaneous effect of distance and un-measured environmental factors, which can be evaluated but the effect is also removed from the model when testing subsequent terms. The effect of species identity was also tested and removed in a similar fashion as the location term, where species identity is included after location in the order dependent ANOVA to evaluate if there is species identity effects on the genetic structure that cannot be attributed to the geographical distribution of the species. After removing direct effects of location and species identity effects of pollution types were evaluated. To test if the effect of pollution type on the genetic structure is influenced by species identity the interaction between species identity and pollution type was also included in the model. The significance of the explanatory variables was assessed using the permutation based ANOVA as implemented in the vegan package (Oksanen et al., 2015) in R 3.2.1 (R Development Core Team, 2015). Each model was corrected for multiple testing using the false discovery rate (FDR) (Benjamini, 2010) online FDR calculator (http://www.sdmproject.com/utilities/?show=FDR) FDR = 0.05.

A different way to perform the cPCoA is to remove the effects of variables, in this case location and species identity, using a condition (Oksanen et al., 2015) which makes it possible to test and visualize the effect of the remaining constraining variable, in this case pollution type. This approach was used to analyze differences between the different pollution types (STP/REF, HAR/REF) in the Baltic Proper.

Three additional constrained ordination analyses were performed, using only sites from Baltic Proper, where differentiation between locations for each pollution type was analyzed separately.

#### Loci associated with the differentiation between STP and REF

To identify alleles at AFLP loci associated with differentiation between the pollution types four approaches were used; two of them, DFDIST (Beaumont & Nichols, 1996) and BayeScan (Foll & Gaggiotti, 2008; Fischer et al., 2011) use an outlier approach, while varSelRF (Diaz-Uriarte, 2007) is a classification method and the Cochran-Mantel- Haenszel is a test for repeated independence (Cochran, 1954; Mantel & Haenszel, 1959; Mantel, 1963). As preliminary analyses including the HAR sites show no signs of genetic differentiation associated with pollution type, the HAR sites were excluded from detailed analyses to identify specific AFLP loci associated with differentiation between pollution types. To avoid identifying loci that were rather associated with genetic differentiation based on geographical differences between the west coast and the Baltic Sea rather than associated with genetic differentiation between STP and REF, only sites from the Baltic Proper were included in these analyses.

DFDIST is a modification of the FDIST approach developed by Beaumont & Nichols (1996) and by implementing the method of Zhivotovsky (1999) to estimate allele frequencies it can be used for dominant markers such as AFLP. DFDIST uses coalescent simulations to generate a null sampling distribution of estimates based on neutral expectations. Loci that do not fit these neutral expectations are identified as putative outliers. Loci with higher *F*_*ST*_ than the expected value may be considered to be under directional selection while loci with lower *F*_*ST*_ values than expected may be considered to be under balancing selection. Here the application Mcheza (Antao & Beaumont, 2011) was applied to run DFDIST. In the DFIST both pairwise (STP and REF from each location) and pollution type-wise (all individuals from each pollution type pooled in the two groups STP and REF) were tested with the settings, simulating the neutral mean distribution of *F*_*ST*_ with 55,000 iterations, a confidence interval of 0.95 and *FDR* = 0.1.

The Bayesian approach implemented in BayeScan (Foll & Gaggiotti, 2008; Fischer et al., 2011) uses the multinomial Dirichlet model with an island model in which the allele frequencies of each subpopulation are correlated through a common migrant gene pool and population-specific and locus-specific components of *F*_*ST*_ coefficients are estimated. The model compares neutral models with models including selection and estimates Bayesian factors (BF) in support of selection over neutrality for each locus (Foll & Gaggiotti, 2008). In BayeScan only the pooled pollution type data was tested (all individuals from each pollution type were pooled in the two groups STP and REF). Twenty pilot runs were conducted with 2,000 iterations, and a burnin of 10,000 iterations resulting in a total of 150,000 iterations. The loci were ranked according to their estimated posterior probability and all loci with a value over 0.91 (corresponding to a Bayes Factor >10) were regarded as outliers.

The package varSelRF (Diaz-Uriarte, 2014) in R 3.2.1 (R Development Core Team, 2015) is based on the ensemble classifier of the decision trees based program, Random Forest (Breiman, 2001), with backwards-variable elimination and the importance spectrum selection based methods. Random Forest was used as an alternative method to identify the loci that contributed most to the differentiation between the pollution types. The principle behind Random Forest is to build a large number of decision trees (a “forest”) using a bootstrap technique (bagging). AFLP loci that are strong predictors of class (here pollution type) will occur in many trees resulting in a higher importance rank (Holliday, Wang & Aitken, 2012; Brieuc et al., 2015). This method is gaining popularity in molecular ecology studies as it has been shown to be useful in the search for loci under selection (Holliday, Wang & Aitken, 2012; Brieuc et al., 2015; Pavey et al., 2015; Laporte et al., 2015; Laporte et al., 2016). In varSelRF different combinations of data were tested, with individuals from each pollution type pooled in the two groups STP and REF and pair wise comparisons of the two pollution types within each location. In varSelRF the parameters were set to mtry = 18 (mtry = the square root of the number of variables i.e., 354) and the ntree = 5,000.

The Cochran-Mantel-Haenszel test for repeated independence (Cochran, 1954; Mantel & Haenszel, 1959; Mantel, 1963) was used to test if there was a consistent difference in proportions of alleles between pollution types over locations. One contingency table for each locus including both pollution type and location was analyzed using package ‘stats’ in R 3.2.1 (R Development Core Team, 2015) (Cochran-Mantel-Haenszel test). To adjust for multiple testing the false discovery rate (FDR) (Benjamini, 2010) the online FDR calculator (http://www.sdmproject.com/utilities/?show=FDR) was used to correct for 354 pairwise comparisons (one/locus), *FDR* = 0.1.

## Results

### AFLP

A total of 396 individuals were used in the analyses and the two primer combinations yielded 354 polymorphic loci (M**CTG**_E**AGC**: 195 loci, M**CTT**_E**ACT**: 159 loci). For error rate analyses 14% of duplicated samples for MCTG_EAGC and 7% of duplicated samples for MCTT_EACT were randomly chosen and yielded a congruent pattern and an error rate of approximately 6% and 7% respectively.

### Gene diversity and genetic differentiation

Gene diversity (*H*_*E*_) for each site and mean gene diversity for each pollution type is presented in Table 1. There was a significant overall partitioning of genetic diversity (*F*_*ST*_ = 0.027, *P*-value <0.01). No significant difference in mean gene diversity between the pollution types was found; including the west coast sites (mean_REF_ = 0.131, mean_STP_ = 0.130, mean_HAR_ = 0.124, ANOVA: *df* = 2, *F* = 0.933, *P*-value = 0.42) and excluding the west coast sites mean_REF_ = 0.128, mean_STP_ = 0.130, mean_HAR_ = 0.124, ANOVA: *df* = 2, *F* = 0.387, *P*-value = 0.690). Significant *F*_*ST*_ pairwise differentiation (after FDR correction, *FDR* = 0.05) was found between the Baltic Proper sites and the west coast sites, and between two pairs of STP and REF sites (ASK_STP/GDA_REF and ASK_STP/KAR_REF) and between one pair of HAR sites (ASK_HAR/KAR_HAR), within the Baltic Proper (Table 2).

When sites were pooled within pollution types, significant genetic differentiation (*F*_*ST*_) between the pollution types STP and REF was observed, both including and excluding the west coast sites (Table 3). No significant genetic differentiation was found between the pollution types HAR and REF either including or excluding the west coast sites (Table 3).

The comparison of genetic differentiation (*F*_*ST*_) between the five locations, ASK, TVA, KAR, GDA and KRI (i.e., 10 pairwise comparisons) for each of the three pollution types showed a significant difference after FDR corrections (*FDR* = 0.05) between all the Baltic Proper locations and KRI and one significant comparison within the Baltic Proper i.e., between ASK and KAR for HAR (Table 3).

**Table 4:** Constrained principal coordinate analyses (cPCoA). Results from a sequence of constrained principal coordinate analyses (cPCoA), were the model terms were tested for significance using order dependent permutation based ANOVA. An alternative way to perform the cPCoA is to remove the effects of variables in the ordination model prior to the ANOVA, in this case location and species identity, using a condition, which makes it possible to test and visualize the independent effect of the remaining constraining variable, in this case pollution type. Each model is based on 354 Amplified Fragment Length Polymorphism (AFLP) loci, using different subsets of the data based on location (BP, Baltic Proper; ALL, Baltic Proper + West Coast; WC, West Coast (i.e., KRI)) and pollution type (STP, sewage treatment plant; REF, reference and HAR, harbor). The significant effects, after false discovery rate (FDR) correction, are indicated in bold (*FDR* = 0.05).

Terms	ALL STP/REF *P*-value	BP STP/REF *P*-value	BP^a^ STP/REF *P*-value	ALL HAR/REF *P*-value	BP HAR/REF *P*-value	BP HAR/REF *P*-value	WC STP/REF *P*-value	WC HAR/REF *P*-value
Location	**0.001** *df* = 4	0.030 *df* = 3	Condition	**0.001** *df* = 4	0.067 *df* = 3	Condition	–	–
Species identity	0.690 *df* = 1	0.682 *df* = 1	Condition	0.938 *df* = 1	0.941 *df* = 1	Condition	0.699 *df* = 1	0.603 *df* = 1
Pollution type	**0.011** *df* = 1	**0.009** *df* = 1	**0.016** *df* = 1	0.634 *df* = 1	0.942 *df* = 1	0.942 *df* = 1	0.823 *df* = 1	0.175 *df* = 1
Species identity : pollution type	0.466 *df* = 1	0.285 *df* = 1	–	0.246 *df* = 1	0.540 *df* = 1	–	–	0.530 *df* = 1

**Figure 2:**
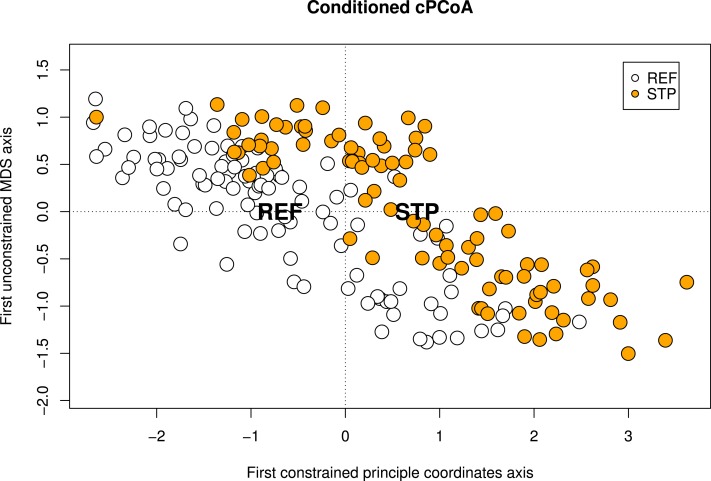
Conditioned constrained principal coordinate analysis (cPCoA). Results from a conditioned constrained principal coordinate analysis (cPCoA), based on Baltic Proper sites. Here, location and species identity are used as conditions and pollution type as constraining variable. The effect of pollution type is significant (*df* = 1, *P*-value = 0.016). The ordination plot shows the effect of pollution type when the variation explained by geographic location and species identity is removed from the ordination. The centroids of each pollution type (STP and REF) are indicated by their abbreviations.

**Notes.**

a Results from this model is plotted in Fig. 2.

### Introgression and species identity

In total, 364 individuals were scored as *M. galloprovincialis* (allele length 124 bp), *M. trossulus* (165 bp) or *M. edulis* (177 bp). Twenty percent of the samples were duplicates and all of the duplicated pairs of samples had the same allele combination. Three individuals contained *M. galloprovincialis* alleles, all in heterozygotes, two from KRI_REF and one from KRI_HAR, but since our analyses are based on variation between and within groups, a group of three is far too small to give reliable results and these three individuals were excluded from subsequent analyses. The remaining individuals were assigned a species identity coded as homozygous *M. trossulus* (2), homozygous *M. edulis* (0) or heterozygotes between the two (1) and species identity was used in subsequent analyses to explore and account for any differences in species identity (Table 1).

To test the species identity effect on the overall genetic structure a constrained principal coordinate analysis (cPCoA) was used, using both the whole data set and a data set excluding the west coast sites. A significant effect of species identity on the genetic structure was found when including the west coast sites (*df* = 1, *F* = 3.90, *P*-value = 0.001), but not when analyzing only the Baltic Proper sites (cPCoA: *df* = 1, *F* = 0.90, *p*-value = 0.739). These results showed a general species identity effect between the west coast and the Baltic Proper, but not within the Baltic Proper.

### Genetic structure

Multivariate constrained principal coordinate analyses (cPCoA) were used to assess the significance of the explanatory variables (constraining variables) location, pollution type and species identity. cPCoA analyses including order dependent, permutation based ANOVAs were conducted on different aspects on the dataset. The cPCoA including all STP and REF sites, using location, species identity and pollution type as constraining variables, was tested with the order dependent ANOVA and showed a significant structuring of the genetic variation both for location and pollution type after FDR correction but not for species identity or the interaction between species identity and pollution type (Table 4). The cPCoA, including STP and REF sites from the Baltic Proper only, using location, species identity and pollution type as constraining variables, showed a significant structuring of the genetic variation after FDR correction for pollution type but not for location, species identity or the interaction between species identity and pollution type (Table 4). The cPCoA including only sites from the west coast (i.e., KRI only) using pollution type as constraint, showed no significant effect of pollution type STP/REF (Table 4).

The conditioned cPCoA, using pollution type, STP and REF as constraint and location and species identity as conditions also showed a significant effect of pollution type on the genetic structure in the Baltic Proper and was used to visualize the effect of pollution type (Table 4, Fig. 2).

In contrast to the STP and REF comparisons, the cPCoA for the HAR and REF data showed no genetic differentiation between the pollution types. When including all sites (both west coast and Baltic Proper) a significant effect (after FDR correction) of location was found, but not for species identity, pollution type or the interaction between the two (Table 4). No effects could be found when analyzing the HAR/REF contrast using the sites from the Baltic Proper (Table 4). The analyses including only sites from the west coast (i.e., KRI only) using pollution type and species identity as constraining variables, showed no significant effects (Table 4). These results indicated that the location effect found when including all sites (both west coast and Baltic Proper) was based on the differentiation between the west coast and Baltic Proper.

To compare the genetic differentiation between the locations for each pollution type independently, three additional cPCoAs were conducted using only sites from Baltic Proper. No significant genetic differentiation between the REF sites (*df* = 3 *F* = 1.05, *P*-value = 0.274) or the STP sites (*df* = 3, *F* = 1.14, *P*-value = 0.080) could be found, while there was a significant difference between the HAR sites (*df* = 4, *F* = 1.23, *P*-value = 0.010).

### Search for specific loci associated with differentiation between the STP and REF

To identify specific marker loci associated with the genetic differentiation between the STP and REF sites four different approaches were used, varSelRF, DFDIST, BayeScan and Cochran-Mantel- Haenszel test. DFDIST and varSelRF were used for both an overall comparison between STP and REF and for pairwise site comparisons. The overall comparison using VarSelRF identified 20 loci and DFDIST 12 significant loci (Table S3). The within location based pairwise comparisons in varSelRF identified between 3 (ASK_STP/REF) to 5 (GDA_STP/REF) loci and DFDIST between 3 (GDA_STP/REF) to 17 (ASK_STP/REF) significant loci (Table S3). In the BayeScan analysis none of the loci had a posterior probability above 0.17, indicating a very low Bayes Factor. However, many of the loci with the highest Bayes Factor (yet not significant) corresponded to loci identified using the other two methods (Table S3). In the Cochran-Mantel- Haenszel test, initially 16 loci were identified as significant, but none of them remained significant after FDR correction. Also here, many of the identified loci (but not significant) corresponded to loci identified using the other methods (Table S3).

## Discussion

Genetic differentiation between sites as measured by *F*_*ST*_ was low within the Baltic Proper (see also Larsson et al., in press). Despite the low over-all differentiation we found that blue mussels populations at STP affected sites had a different genetic composition than populations at reference sites, suggesting non-random settling and/or survival at STP sites. This effect of sewage water is in line with earlier findings in bat stars (*Patiria miniata*) where a significant correlation between storm- and wastewater and genetic structure was found (Puritz & Toonen, 2011). Puritz & Toonen (2011)’s study and the present study are among the first studies to show genetic differentiation associated with pollution from sewage treatment plants on wild populations. In contrast, we did not find any results suggesting a similar pattern between the HAR and REF sites. However, we found that mussel populations from Baltic Proper HAR sites were significantly genetically divergent from each other, while REF and STP sites were not (as shown by the pollution type based cPCoA). This suggests that there is a divergent effect of pollution from harbors, while the pollution effect from sewage treatment plants on the genetic composition of blue mussel populations seems to act in the same direction at the investigated sites.

Anthropogenic habitat changes can lead to increase or decrease in gene diversity (*H*_*E*_). Multiple studies of marine organisms have shown a range from significantly lower *H*_*E*_ (Ross et al., 2002; Gardeström et al., 2008; Fratini et al., 2008; Puritz & Toonen, 2011), no difference (McMillan et al., 2006; Lind & Grahn, 2011),and higher *H*_*E*_ (Štambuk et al., 2013) in anthropogenically affected habitats, suggesting that the life history and biogeography of the study organism as well as the type and concentration of the contaminants influences the effects on gene diversity. In the present study, we did not find any differences in gene diversity (*H*_*E*_) between STP and REF sites or between HAR and REF sites, most likely reflecting high connectivity and high gene flow among the sites.

### Genetic differentiation in blue mussels between reference sites and sewage treatment plant sites in the Baltic Proper

The major genetic features of blue mussels in the sampled area were the expected pronounced genetic differentiation between the west coast and the Baltic Sea, and the weak differentiation within the Baltic Proper caused by strong gene flow. Genetic differentiation in the blue mussel between the west coast and the Baltic Sea has previously been identified in several studies (Johannesson & Andre, 2006; Wennerström et al., 2013; Larsson et al., in press). In the present study, the pairwise *F*_*ST*_ analyses (Table 2) confirm this geographical pattern. The present study also identified a higher frequency of *M. edulis* alleles in the west coast sites (Table 1), and an overall significant effect of species identity on the genetic structure, however the effect of species identity on the genetic structure was not significant when geographical location was accounted for in the analyses, indicating that differences other than species origin might be present similar to what was found in Larsson et al. (in press). Within the Baltic Proper no effect of species identity on the genetic structure was found, also similar to what was found in Larsson et al. (in press). The current study shows a weak over-all genetic differentiation within the Baltic Proper as shown by very few significant pairwise *F*_*ST*_ values (Table 2). When analyzing genetic differentiation between locations within each pollution type separately, no significant differentiation between locations was found within the pollution types STP and REF as shown both by the location based *F*_*ST*_ analyses (Table 3) and by the cPCoAs. These results confirm the earlier findings of low differentiation between locations in the Baltic Proper, due to high oceanographic connectivity and strong gene flow (see Larsson et al., in press).

Despite the low general genetic differentiation within the Baltic Proper a significant differentiation between pollution type STP and REF was detected. This was shown by several analyses such as the cPCoA (Table 4, Fig. 2), the pollution type based *F*_*ST*_ analyses, and in two significant pairwise *F*_*ST*_ analyses (Table 2). This effect seems to be restricted to the Baltic Proper as no significant difference between STP and REF on the west coast was found (Table 4). Sewage effluents have also been shown to be associated with population genetic effects in other systems. Puritz & Toonen (2011) detected that genetic structure of the bat star (*P. miniata*) correlated with major sources of sewage effluents and storm water. The bat star has a similar life history to the blue mussel with large populations and strong gene flow caused by larval dispersal over large distances. Despite this strong gene flow Puritz & Toonen (2011) were able to show that differences between sites (i.e., pairwise *F*_*ST*_) could be explained by sewage water causing a barrier to larval dispersal.

The significant differentiation between STP and REF sites despite strong gene flow is in line with studies finding significant genetic differentiation among populations when subjected to high gene flow (Nosil, Funk & Ortiz-Barrientos, 2009; Lamichhaney et al., 2012; Gagnaire et al., 2012; Ulrik et al., 2014; Laporte et al., 2016; Guo, Li & Merilä, 2016). Taking into account the generally low genetic differentiation within the Baltic Proper and the nested study design, evolutionary forces like restricted gene flow, directional gene flow and genetic drift are considered less likely to have caused the observed genetic differentiation between STP and REF. The most likely evolutionary force acting on these populations is selection. As we do not have any data on local recruitment over generations, we conservatively describe the observed pattern as a within-generation footprints of local directional selection, in parallel with the findings in the American eel (*A. anguilla*) and European eel (*A. rostrata*) (Gagnaire et al., 2012; Ulrik et al., 2014; Laporte et al., 2016).

The AFLP method, employed in the present study, has previously been shown to identify specific marker loci linked to adaptive variation among habitats (Williams & Oleksiak, 2008; Nosil, Funk & Ortiz-Barrientos, 2009; Lind & Grahn, 2011). This possibility was explored by using four different analyses. The combination of analyses identified a large number of loci (Table S3), however only a few loci were verified across methods and combinations of data set (i.e., pair-wise and pooled STP and REF). No significant loci were identified using BayeScan and loci identified using CMH were not significant after FDR correction. The identification of only a few specific loci associated with the differentiation may be due to small allelic changes among many co-varying loci rather than pronounced changes at a few loci of large effects (Laporte et al., 2016). To further explore such polygenic selection, a high resolution sequencing method (e.g., RAD sequencing or genome sequencing) in combination with approaches not based on the hitch-hiking model would be needed (Laporte et al., 2016).

The anonymous nature of AFLP and the suggested polygenic nature of STP effects make it difficult to imply any direct mechanisms behind the observed differentiation. Cage experiments exposing adult mussels outside sewage treatment plants have shown to include several direct (pharmaceuticals and endocrine disrupting compounds) and indirect stressors (nutrients load-induced algae blooms and/or temporal anoxia/hypoxia), likely reflecting the complexity of sewage effluents. The stressors have included immunological (Akaishi et al., 2007), genotoxic and lysosomal responses (Turja et al., 2015) but also endocrine disruptive effects such as feminization in freshwater mussels (*Elliptio complanata*) (Gagné et al., 2011) and up-regulation of vitellogenin in males in both freshwater mussels *Elliptio complanata* (Bouchard et al., 2009; Gagné et al., 2011), and in marine mussels *Mytilus galloprovincialis* (De los Ríos et al., 2013). The experiments described above were conducted on transplanted adult individuals (De los Ríos et al., 2013; Turja et al., 2015) and in this study the mussels examined were likely exposed to pollutants throughout their life, and it is therefore difficult to imply at which life stages of the blue mussels the putatively local selection occurs. Toxicity studies have shown that larvae are more sensitive towards many pollutants when compared to adults (Beiras & Bellas, 2008), and as Puritz & Toonen (2011) suggest that waste and storm water act as barrier to larval dispersal, it is therefore plausible that STP effluent effects occur at early life stages.

As the blue mussels in the Baltic Proper show a strong introgression pattern (Väinölä & Strelkov, 2011) the effect of species identity on the genome wide genetic structure was explored, while possible effects of species differences was controlled for when analyzing pollution type effects. In this study, species identity, based on the Glu-5′ marker, was not associated with genetic structure in any of the analyses when the effect of large-scale geographical distances were accounted for. The lack of association between species identity and genetic structure in the Baltic Proper supports the presence of strong introgression in the Baltic Proper (Larsson et al., in press). These results also show that species identity is not an important part of the pollution type effects on genetic differentiation. If species identity was an important factor in the STP effects on genetic structure, a significant interaction between species and STP would have been observed. It is, however, possible that the hybridization status of each individual was not fully covered by the Glu’-5 marker and that other markers based on other parts of the genome may give a discrepant pattern (see Kijewski et al., 2006; Kijewski et al., 2011). To study introgression patterns in detail, a more comprehensive full genome scan including pure *M. edulis* and *M. trossulus* as reference individuals is needed but this was beyond the scope of the present study.

### Genetic divergence between harbor sites (HAR) in the Baltic Proper

In contrast to the genetic differentiation found between STP and REF sites, we did not find any results suggesting a congruent effect on the genetic differentiation between the HAR and REF sites. However, the cPCoA show significant differentiation among HAR sites in the Baltic Proper. This pattern may be interpreted as a signature of divergent local selection among HAR sites and is in contrast to a study of Mediterranean mussels where no significant genetic differentiation between harbor sites was found (Štambuk et al., 2013). A possible explanation may be that the harbors each have a diverse pollution regime, and the selection pressure therefore differs between them. The results indicate that there may be differences in the pollution regimes between the harbor types (naval port, oil harbor and ferry ports). Another factor that could affect differentiation between harbor populations is shipping. All studied harbors are connected to inter- and intra- Baltic shipping routes, so it is possible that harbor populations may be differentiated via introductions of individuals from different areas in- and outside the Baltic Sea.

Similar to what was found for the STP pollution type, no significant effects of species identity were found, and again this result indicates that species identity is not an important part of the pollution type effect on genetic differentiation.

## Conclusions

In conclusion, this study identified genetic differentiation in the Baltic Sea blue mussel associated with exposure to sewage treatment plant effluents. This study adds to the growing body of literature showing genetic effects associated with exposure to sewage treatment plant effluents on wild populations. Blue mussels from HAR sites were more genetically divergent than mussels from STP and REF sites, indicating possible divergent selection caused by different pollution regimes in different harbors or by inter- and intra- Baltic shipping routes. This putative within-generation footprint of local selection in blue mussels despite extensive gene flow through pelagic larval dispersal suggests that in other organisms, with other life history characters that promote e.g., local recruitment, pollution may direct evolutionary processes to an even higher degree. The results imply that evolutionary effects of pollution on wild populations are important for ecosystem function and biodiversity conservation.

##  Supplemental Information

10.7717/peerj.2628/supp-1 Table S1 Process type and pollution data from the sampled STP sites Name of Sewage treatment plant, size of the municipal area (no. of persons and person equivalents), type of waste water process. Record of eutrophication (N and P), main pollutants (i.e., heavy metals in the effluent water). NA = no available data.

10.7717/peerj.2628/supp-2 Table S2 Traffic and pollution data from the sampled HAR sites Name and type of harbor, traffic (number of boats/year). Record of main pollutants (i.e., heavy metals in the sediment as site). NA = no available data.

10.7717/peerj.2628/supp-3 Table S3 Loci identified to be associated with the differentiation between the STP and REF sites in the Baltic Proper, using varSelRF (pairwise and pooled STP and REF sites), DFDIST (pairwise and pooled STP and REF sites), BayeScan (pooled STP and REF sites) and Cochran-Mantel- Haenszel test

10.7717/peerj.2628/supp-4 Supplemental Information 1 Reference list for supplementary material

10.7717/peerj.2628/supp-5 Data S1 Raw Data The raw data presented including all individuals analyzed, with sample name, sampling site, sampling location, pollution type, species identity coded as Mytilus trossulus (T), heterozygotes (H), Mytilus edulis (E), species identity coded as Mytilus trossulus (2), heterozygotes (1), Mytilus edulis (0), AFLP data with each marker coded with name and number. The data is divided into subsets, ALL representing the total data set, BP representing a subset of all individuals sampled from the Baltic Proper. STP_REF_ representing a subset of all individuals sampled from the reference sites (REF) and sewage effluent affected sites (STP) in the total study area (ALL) and the Baltic Proper (BP). HAR_REF_ representing a subset of all individuals sampled from the reference sites (REF) and harbor sites (HAR) in the total study area (ALL) and the Baltic Proper (BP).

10.7717/peerj.2628/supp-6 Supplemental Information 2 R code

## Additional Information and Declarations

### Data Availability

The following information was supplied regarding data availability: The raw data and code has been supplied as Supplementary Files.
