# Supplementary material for: Sewage treatment plant associated genetic differentiation in the blue mussel from the Baltic Sea and Swedish west coast
Source: PeerJ. 2016 Oct 27;4:e2628. doi: 10.7717/peerj.2628 (PMC5088577; doi:10.7717/peerj.2628)
Supplement: Table S1 — Name of Sewage treatment plant, size of the municipal area (no. of persons and person equivalents), type of waste water process. Record of eutrophication (N and P), main pollutants (i.e., heavy metals in the effluent water). NA = no available data. [file peerj-04-2628-s001.docx]

| **SITE** | **ASK_STP** | **TVA_STP** | **KAR_STP** | **GDA_STP** | **KRI_STP** |
| --- | --- | --- | --- | --- | --- |
| **Name** | **Himmerfjärden** | **Stormossen** | **Koholmen** | **Orlow** | **Långevik** |
| Number of persons | 306 500 | 9 000 | 42 000 | 360 000 | 7 900 |
| Industry waste ^(population equivalents, pe)^ | 20 000 | 60 412 m3/a | 7000 |  | 2 400 |
| Type of process | Grit 2 mm | Grit 3 mm | Grit |  | Grit 2 mm |
|  | Sand grit (fat reducer) | Sand grit (fat reducer) | Sand grit (fat reducer) |  | Sand grit (fat reducer) |
|  | Sedimentation | Sedimentation | Biological treatment: aeration, denitrification, nitrification, deoxidification |  | Aeration |
|  | Biological treatment: aeration, nitrification | Biological treatment: aeration | Sedimentation |  | Pre-sedimentation |
|  | Sedimentation | Sedimentation | Chemical treatment flocking |  | Biological treatment: nitrification and denitrification |
|  | Denitrification | Chemical treatment Acti-Flow | Sedimentation |  | Chemical treatment flocking |
|  | Sandfilter |  | Sandfilter |  | Final sedimentation |
| N total mean/year (mg/l) | 7.8 | 20 | 8.2 |  | 12 |
| P total mean/year (mg/l | 0.32 | 0.54 | 0.18 |  | 0.2 |
| BOD7 total mean/year (mg/l) | 6.9 | 7.4 | 2.50 |  | 8 |
| N total/year (T) | 380* | NA | 52 | 100.83 | 28.5 |
| P total/year (T) | 16* | NA | 1.1 | 0.45 | 0.5 |
| BOD7 (T) | NA | NA | 16 |  | 19.7 |
| Deep outlet (m) | 25 (10) | NA | 20 | 10 | 24 |
| Hg (μl/l) | < 0.10 | NA | 0.055 | NA | 0.05 |
| Cd (μl/l) | < 0.10 | NA | 0.058 | NA | 0.01 |
| Pb (μl/l) | < 0.50 | NA | 0.11 | NA | 0.34 |
| Cu (μl/l) | 11.7 | NA | 9.4 | NA | 140 |
| Zn (μl/l) | 14.3 | NA | 29 | NA | 30 |
| Cr (μl/l) | 1 | NA | 1.2 | NA | 0.5 |
| Ni (μl/l) | 4.3 | NA | 2.6 | NA | 7 |
| Al (μl/l) | NA | NA | 220 | NA | NA |
| Fe (μl/l) | NA | NA | NA | NA | NA |
| Year ^Reference^ | 2013^1)^ | 2010^2)^ | 2012^3)^ | 2011^4)^ | 2013^5)^ |

*Larsson et al. 2012, ^1)^ Söhr, 2013, ^2)^ Töyrylä, 2012, ^3)^ Strand et al. 2012, ^4)^ Technical report – 2011, Institute of Oceanography, University of Gdańsk, ^5)^ Lysekils kommun, 2013.
