# Supplementary material for: Sewage treatment plant associated genetic differentiation in the blue mussel from the Baltic Sea and Swedish west coast
Source: PeerJ. 2016 Oct 27;4:e2628. doi: 10.7717/peerj.2628 (PMC5088577; doi:10.7717/peerj.2628)
Supplement: Table S2 — Name and type of harbor, traffic (number of boats/year). Record of main pollutants (i.e., heavy metals in the sediment as site). NA = no available data. [file peerj-04-2628-s002.docx]

| **SITE** | **ASK_HAR** | **TVA_HAR** | **KAR_HAR** | **GDA_HAR** | **KRI_HAR** |
| --- | --- | --- | --- | --- | --- |
| **Name** | **Nynänshamn harbor** | **Hanko harbor** | **Karlskrona navy harbor** | **Gdansk harbor** | **Preem oil harbor** |
| Traffic (boats/year) | NA | NA | NA | NA | 90 |
| **Environmental data** |  |  |  |  |  |
| As (mg/kg) | 10.9 | NA | 10.4 | NA | NA |
| Ba (mg/kg) | NA | NA | 306 | NA | NA |
| Be (mg/kg) | NA | NA | 0.757 | NA | NA |
| Cd (mg/kg) | 0.8 | NA | 1.26 | ^4.9 | NA |
| Co (mg/kg) | 9.34 | NA | 6.59 | ^10 | NA |
| Cr (mg/kg) | 48.2 | NA | 32.3 | ^89 | NA |
| Fe (mg/kg) | 109 | NA | 17200 | ^31 | NA |
| Cu (mg/kg) | NA | NA | 185 | NA | NA |
| Hg (mg/kg) | 1.98 | NA | 2.55 | ^1.1 | NA |
| Mo (mg/kg) | 6.54 | NA | 1.92 | ^1.3 | NA |
| Mn (mg/kg) | NA | NA | 200 | NA | NA |
| Ni (mg/kg) | 29.4 | NA | 21.4 | ^12 | NA |
| Pb (mg/kg) | 1050 | NA | 303 | ^35 | NA |
| Sn (mg/kg) | NA | NA | 1.76 | NA | NA |
| V (mg/kg) | 60.3 | NA | 30.7 | ^84 | NA |
| Zn (mg/kg) | 169 | NA | 419 | ^95 | NA |
| EPA-PAH (16) (mg/kg TS) | <0.4 | NA | 70 | *195ng/l | NA |
| PCB (7)(mg/kg TS) | <0.035 | NA | 0.335 | ^0.00004 | NA |
| TBT (ug/kg TS) | 5.1 | NA | NA | NA | NA |
| Year | 2007^6)^ | - | 2009^7)^ | 2011^8)^ | - |
| Sampling depth (m) | 13.5 | NA | NA | NA | NA |
| ^1)^ Land, 2007 | * Bottom water |  |  |  |  |
| ^2)^ Bard et al. 2009 | ^Sediment |  |  |  |  |
| ^3)^ Rogowska, 2011 |  |  |  |  |  |
