# Supplementary material for: Sewage treatment plant associated genetic differentiation in the blue mussel from the Baltic Sea and Swedish west coast
Source: PeerJ. 2016 Oct 27;4:e2628. doi: 10.7717/peerj.2628 (PMC5088577; doi:10.7717/peerj.2628)
Supplement: Supplemental Information 1 [file peerj-04-2628-s004.docx]

Reference list for Supplementary material

Bard J., Tornberg K., Björinger P., Hjort T., Carlsson A., Wodschow N. Försvarsmakten Miljöprövningsenheten. Söderstierna – Lindholmen, Marinbasen Karlskorna. Kompletterande undersökning, fördjupad riskbedömning och åtgärdsutredning. 2010. NIRAS Johan Héllden AB, Linköping (ref: 80849-1)

Institute of Oceanography. 2011. Technical report. University of Gdańsk, Gdansk.

Land, M. 2007. Miljöteknisk undersökning av sediment vid Norvik, Nynäshamns kommun. WSP Environmental Mark och Vatten Stockholm (ref: 10097492)

Larsson, U., Nyberg, S., Höglander, H., Sjösten, A., Sandberg, M., Walve, J. 2012. Himmerfjärden, rapport till SYVAB.

Lysekils kommun, 2013. Miljörapport för år 2013, Långeviks avloppsreningsverk 2013, Lysekils kommun, LEVAi Lysekil AB.

Rogowska J., 2011. Wpływ wraków na środowisko na przykładzie s/s Stuttgart. Politechnika Gdańska, Wydział Chemiczny, Katedra Chemii analitycznej, pp. 151

Söhr, S., 2013. Miljörapport 2013, syvab Himmerfjärdsverket. Syvab.

Strand, M. 2012. Miljörapport för år 2012, Koholmens avloppsreningsverk

Karlskrona kommun. Tekniska förvaltningen, Karlskrona kommun.

Töyrylä, T., 2012. Hangö Stad, utvecklingsplan för vattentjänsterna. Ramboll, Helsinki.
