# Supplementary material for: Sewage treatment plant associated genetic differentiation in the blue mussel from the Baltic Sea and Swedish west coast
Source: PeerJ. 2016 Oct 27;4:e2628. doi: 10.7717/peerj.2628 (PMC5088577; doi:10.7717/peerj.2628)
Supplement: Supplemental Information 2 [file peerj-04-2628-s006.pdf]

Supplementary R code for analyses Larsson et al.

####DIFFERENCES IN H_E TESTED WITH ANOVAS in Rcmdr##

```
library(Rcmdr)
```

##H_E derived from AFLP-surv see table 1###

```
data <-read.table("infile.txt",header=T,sep="\t",dec=".",
na.strings="NA",row.names="FST")

LM_HE <- lm(HE ~ POLLUTION_TYPE, data=data)
summary(LM_HE)
Anova(LM_HE, type="II")
```

####SPECIES EFFECT ON GENETIC STRUCTURE

###Using individuals from the raw data set in different combinations, here exemplified using ALL sites##

```
library(vegan)

musslor<-read.table ("RAWDATA_AFLP_Larsson_etal.txt",header=T,
sep="\t",dec=",",na.strings="NA")

musslor<-na.omit(musslor)

mdata<-musslor[1:6]
maflp<-musslor[7:360]
names(mdata)
names(maflp)

distt<- vegdist(maflp,method='jaccard')
ecapvar<-capscale(distt~ SPECIES_IDENTITY, data=mdata)
anova(ecapvar, max.perm=1000,by="terms")
```

###GENETIC STRUCTURE cPCoA UISNG LOCATION, SPECIES IDENTITY, POLLUTION_TYPE AND THE INTERACTION BETWEEN SPECIES:POLLUTION_TYPE AS CONSTRAINTS###

###Using individuals from the raw data set in different combinations, here exemplified using BP sites, STP/REF##

```
library(vegan)

musslor<-read.table ("RAWDATA_AFLP_Larsson_etal.txt",header=T,
```

```
sep="\t",dec=",",na.strings="NA")

musslor<-na.omit(musslor)

mdata<-musslor[1:6]
maflp<-musslor[7:360]
names(mdata)
names(maflp)

distt<- vegdist(maflp,method='jaccard')
ecapvar<-capscale(distt~LOCATION+ SPECIES_IDENTITY
+POLLUTION_TYPE+ SPECIES_IDENTITY:POLLUTION_TYPE, data=mdata)
anova(ecapvar, max.perm=1000,by="terms")

###GENETIC STRUCTURE cPCoA UISNG LOCATION, SPECIES IDENTITY AS
CONDTIONS AND POLLUTION TYPE AS CONSTRAINTS###

library(vegan)

musslor<-read.table ("RAWDATA_AFLP_Larsson_etal.txt",header=T,
sep="\t",dec=",",na.strings="NA")

musslor<-na.omit(musslor)

mdata<-musslor[1:6]
maflp<-musslor[7:360]
names(mdata)
names(maflp)

distt<- vegdist(maflp,method='jaccard')
ecapvar<-
 capscale(distt~POLLUTION_TYPE,Condition(LOCATION+SPECIES_IDENTITY)
 , data=mdata)

anova(ecapvar, max.perm=1000,by="terms")

##PLOT

plot(ecapvar)
plot<-ordiplot(ecapvar,type="none")
pocform<-factor(mdata$POLLUTION_TYPE,labels=c("21","21"))
coll<-factor(mdata$POLLUTION_TYPE,labels=c("white","orange"))
collda=as.character(coll)
fige<-as.numeric(as.character(pocform))
pocplot<-ordiplot(ecapvar,type="none",main="Conditioned cPCoA",xlab="First
 constrained principle coordinates axis",
ylab="First unconstrained MDS axis")

points(plot,"sites",pch=fige,col="black",bg=collda,cex=2.0)
text(plot,"centroids",labels=with(mdata,levels(POLLUTION_TYPE)),col="black",
```

```
 bg="white", cex=1.5, font=2)

legend (locator(1), c("REF","STP"),
pch=c(21,21),cex=1.0,pt.cex=1.0,pt.bg=c("white","orange"), col="black")



##### GENETIC STRUCTURE cPCoA (SITES AS CONSTRAINT) BALTIC
PROPER SITES FROM EACH POLLUTION TYPE SEPARATELY####

##Using individuals from each pollution type and only Baltic proper sites
seperatly####

library(vegan)

musslor<-read.table ("RAWDATA_AFLP_Larsson_etal.txt",header=T,
sep="\t",dec=",",na.strings="NA")

musslor<-na.omit(musslor)

mdata<-musslor[1:6]
maflp<-musslor[7:360]
names(mdata)
names(maflp)

distt<- vegdist(maflp,method='jaccard')
ecapvar<-capscale(distt~SITE, data=mdata)
anova(ecapvar, max.perm=1000,by="terms")

####LOCI ASSOCIATED WITH GENETIC DIFFERENTIATION varSelRF####

library(varSelRF)

###Using only STP and REF individuals from the raw data###
mussel<-read.table("RAW_DATA_Larssonetal", header=T,sep="\t", dec=",")
names(mussel)

varSelRF(mussel[3:356],mussel$POLLUTION_TYPE,c.sd = 1, mtryFactor = 18,
ntree = 5000,
        ntreeIterat = 2000, vars.drop.num = NULL, vars.drop.frac = 0.2,
        whole.range = TRUE, recompute.var.imp = FALSE, verbose = FALSE,
        returnFirstForest = TRUE, fitted.rf = NULL, keep.forest = FALSE)
```
